# CNP-miR146a Decreases Inflammation in Murine Acute Infectious Lung Injury

**DOI:** 10.3390/pharmaceutics15092210

**Published:** 2023-08-26

**Authors:** Alyssa E. Vaughn, Tanner Lehmann, Christina Sul, Alison M. Wallbank, Bailey D. Lyttle, James Bardill, Nana Burns, Anisha Apte, Eva S. Nozik, Bradford Smith, Christine U. Vohwinkel, Carlos Zgheib, Kenneth W. Liechty

**Affiliations:** 1 Laboratory for Fetal and Regenerative Biology, Department of Surgery, University of Colorado Denver and Children’s Hospital Colorado, Aurora, CO 80045, USA; 2 Cardiovascular Pulmonary Research Laboratories and Pediatric Critical Care Medicine, Department of Pediatrics, University of Colorado Denver, Aurora, CO 80045, USA; 3 Department of Bioengineering, University of Colorado Denver, Aurora, CO 80045, USA; 4 Laboratory for Fetal and Regenerative Biology, Department of Surgery, University of Arizona Tucson College of Medicine and Banner Children’s at Diamond Children’s Medical Center, Tucson, AZ 85721, USA

**Keywords:** bioactive nanoparticle therapeutic, infectious lung injury model, acute respiratory distress syndrome, cerium oxide nanoparticles (CNP), microRNA-146a (miR146a)

## Abstract

Acute respiratory distress syndrome (ARDS) has approximately 40% in-hospital mortality, and treatment is limited to supportive care. Pneumonia is the underlying etiology in many cases with unrestrained inflammation central to the pathophysiology. We have previously shown that CNP-miR146a, a radical scavenging cerium oxide nanoparticle (CNP) conjugated to the anti-inflammatory microRNA(miR)-146a, reduces bleomycin- and endotoxin-induced acute lung injury (ALI) by decreasing inflammation. We therefore hypothesized that CNP-miR146a would decrease inflammation in murine infectious ALI. Mice were injured with intratracheal (IT) MRSA or saline followed by treatment with IT CNP-miR146a or saline control. Twenty-four hours post-infection, bronchoalveolar lavage fluid (BALF) and whole lungs were analyzed for various markers of inflammation. Compared to controls, MRSA infection significantly increased proinflammatory gene expression (IL-6, IL-8, TNFα, IL-1β; *p* < 0.05), BALF proinflammatory cytokines (IL-6, IL-8, TNFα, IL-1β; *p* < 0.01), and inflammatory cell infiltrate (*p* = 0.03). CNP-miR146a treatment significantly decreased proinflammatory gene expression (IL-6, IL-8, TNFα, IL-1β; *p* < 0.05), bronchoalveolar proinflammatory protein leak (IL-6, IL-8, TNFα; *p* < 0.05), and inflammatory infiltrate (*p* = 0.01). CNP-miR146a decreases inflammation and improves alveolar–capillary barrier integrity in the MRSA-infected lung and has significant promise as a potential therapeutic for ARDS.

## 1. Introduction

Acute respiratory distress syndrome (ARDS) is a life-threatening illness in both adults and children. ARDS exerts a substantial disease burden with significant mortality estimated to be approximately 38.5% in adults [1,2] and 18–27% in children [3] and significant morbidity for long-term survivors with about 50% of survivors unable to return to work within a year of diagnosis [4,5]. ARDS is also associated with extensive financial burden to both individuals and the healthcare system [6]. Unfortunately, despite extensive research in search of therapeutic options for ARDS, current management remains largely limited to supportive care focusing on lung-protective ventilation and low-volume fluid resuscitation [7,8,9].

Acute lung injury (ALI) that progresses to ARDS can result from a variety of etiologies, and pneumonia is associated with approximately 60% of cases [10]. Regardless of the inciting cause, both ALI and ARDS share the pathogenesis of overwhelming and unrestrained inflammation and oxidative stress [11,12] resulting in alveolar–capillary barrier breakdown [13,14] with pulmonary edema and severe hypoxia, which in turn promote downstream lung fibrosis [15]. The dysregulated inflammatory response seen in ARDS is mediated by release of proinflammatory cytokines and chemokines and leads to a self-reinforcing feedback loop of inflammation that can be life-threatening [16]. Inhibition of this inflammatory cascade offers a potential therapeutic target for the treatment of ALI and ARDS.

We have previously shown that a novel therapeutic CNP-miR146a, the product of conjugation of cerium oxide nanoparticles (CNP) to microRNA-146a (miR146a), can decrease inflammation and oxidative stress with subsequent improvement in both lung structure and function in both bleomycin-induced fibrotic lung injury and lipopolysaccharide (LPS)-induced sterile inflammatory lung injury models [17,18,19]. However, the effects of CNP-miR146a on lung injury caused by infectious pneumonia were unknown. CNPs are metal oxides which have a mixed valency that allows for dynamic binding of reactive oxygen species (ROS) [20] and augments the natural ability of cells to handle ROS. CNPs also function as a novel delivery system for microRNAs and allow for stable delivery of targeted microRNAs to the tissue [21]. MicroRNAs are small, noncoding RNAs that play a crucial role in the regulation of gene expression. Previous studies have identified miR146a as a “molecular brake” on inflammation, specifically functioning to inhibit tumor-necrosis-factor-receptor-associated factor 6 (TRAF6) and interleukin-1 receptor associated kinase-1 (IRAK1), which then decrease activation of the nuclear factor kappa-light-chain-enhancer of activated B cells (NF-κB) inflammatory pathway. NF-κB downregulation ultimately results in decreased levels of proinflammatory cytokines such as interleukin (IL)-6, IL-8, IL-1β, and tumor necrosis factor alpha (TNFα) [22,23]. 

Given the large proportion of ARDS cases caused by pneumonia and that infectious pneumonia is a different pathophysiologic entity than chemical or sterile inflammatory lung injury, the current study probes the effects of CNP-miR146a on pulmonary inflammation in a murine model of infectious lung injury. Additionally, a single animal model is unable to fully recapitulate all of the features of human ARDS [13], therefore providing further reason to evaluate the efficacy of CNP-miR146a as a potential therapeutic for ARDS in a model of infectious pneumonia. Methicillin-resistant *Staphylococcus Aureus* (MRSA) is a serious pathogen resistant to certain antibiotics and can cause significant pulmonary infections in hospitalized or immunocompromised patients [24,25]. Recruited inflammatory cells are key in initiating inflammation in response to MRSA pneumonia and the release of ROS, cytokines, proteases, and many inflammatory mediators which contribute to the dysregulated inflammation seen in ALI and ARDS [26,27]. Based on this information, we determined MRSA pneumonia to be an acceptable infectious murine model of ALI with which to evaluate our novel therapeutic. We hypothesized that intratracheal (IT) administration of CNP-miR146a following MRSA-induced ALI would mitigate the inflammatory response in the lung by decreasing proinflammatory gene expression and inflammatory cell infiltrate and improving alveolar–capillary barrier integrity. 

## 2. Materials and Methods

### 2.1. Development of CNP-miR146a

CNPs were synthesized via chemical hydrolysis and then conjugated to miR146a using 1′-1′ carbonyldiimidazole (CDI) chemistry to covalently bind the miR146a amino group to the CNP hydroxyl group using an amine linker group, as previously described [28,29]. The amine linker group allows space between the miRNAs and the central CNP. The conjugated CNP-miR146a was then prepared for IT dosing by diluting it to 0.5 ng/kg (or approximately 0.01 ng/mouse) in phosphate buffered saline (PBS). 

### 2.2. MRSA Infectious Lung Injury Animal Model

All animal studies were approved by the Institutional Animal Care and Use Committee (IACUC, protocol #00128) at the University of Colorado Denver|Anschutz Medical Campus. Care for animals was conducted by trained veterinarians and technicians according to the National Institutes of Health Guide for the Care and Use of Laboratory Animals. All mice were maintained in standard housing for at least one week prior to experimentation to allow for acclimation to ambient Denver atmosphere (1600 m altitude). Animal numbers were minimized where possible, for example, by utilizing the same animals to assess bronchoalveolar lavage fluid (BALF) as well as lung tissue for biochemical evaluation. However, separate lung tissue harvests were required for biochemical evaluation and histologic analysis due to different methods of tissue processing. 

Eight- to twelve-week-old C57BL/6 male and female mice (Jackson Laboratory) were randomly assigned to receive 30 µL IT PBS or 1 × 10^8^ colony forming units (CFUs) of methicillin-resistant *Staphylococcus Aureus* (Sa; clinical strain USA300 LAC, from M.G. Bowden, University of Houston, Houston, TX, USA) in 30 µL PBS by IT instillation under 1.5% isoflurane anesthesia and 0.21 fraction of inspired oxygen. After four hours, a cohort of MRSA-infected mice were treated with 0.5 ng/kg or 0.01 ng/mouse IT CNP-miR146a dissolved in PBS equaling a volume of 50 µL while control mice were administered 50 µL IT PBS. CNP-miR146a concentration was assessed by Quant-iT™ (ThermoFisher Scientific Quant-iT™ microRNA Assay Kit, Waltham, MA, USA) to determine miR146a concentration and inductively coupled plasma mass spectrometry (ICP-MS) to determine cerium concentration. Mice were euthanized by carbon dioxide gas at 24 h post-inoculation followed by thoracotomy, bronchoalveolar lavage, and harvesting of lung tissue to evaluate the parameters below. A previous study demonstrated the conjugate therapeutic (CNP-miR146a) is more effective in the treatment of ALI than either individual component alone (CNP or miR146a mimetic) [17], therefore we focused the current investigation on a comprehensive evaluation of the effects of the combined therapeutic to optimize animal use. The following groups were included in the present study: control (PBS + PBS), injured and untreated (MRSA + PBS), and injured and treated (MRSA + CNP-miR146a). 

### 2.3. Proinflammatory Gene Expression Measurement

Whole lung tissue was excised at 24 h post-inoculation, snap frozen in liquid nitrogen, and stored at −80 °C (n = 6–7 per group). Whole bilateral lungs were homogenized in Qiazol (Qiagen) in accordance with manufacturer instructions. Isolated RNA concentration was quantified with Nanodrop (ThermoFisher Scientific), treated with DNase 1 (ThermoFisher), and converted to cDNA (Applied Biosystems RT kit). The cDNA was then amplified by reverse transcriptase with a MasterCycler Gradient Thermal Cycler (Eppendorf, Framingham, MA, USA). Real-time quantitative polymerase chain reaction (RT-qPCR) was performed using a CFX96 Touch Real-Time PCR Detection System (Bio-Rad, Hercules, CA, USA) for proinflammatory genes IRAK1, TRAF6, NF-κB, IL-6, macrophage inflammatory protein-2 (MIP-2) (the murine analog of human IL-8), TNFα, and IL-1β, which were normalized to the housekeeper gene glyceraldehyde 3-phosphate dehydrogenase (GAPDH). All RT-qPCR primers utilized published sequences which are commercially available (TaqMan Primers, ThermoFisher Scientific). For evaluation of miRNA levels between groups, isolated RNA samples were diluted to 5 ng/µL, converted to U6 and miR146a cDNA (Applied Biosystems RT kit, ThermoFisher Scientific), and amplified by reverse transcriptase amplification. RT-qPCR was then performed for miR146a and normalized to the housekeeper gene U6. 2.4. Bronchoalveolar Lavage Fluid Proinflammatory Protein Concentration.

BALF was collected at 24 h post-infection by performing a tracheostomy through which 0.8 mL ice-cold PBS was twice instilled and gently suctioned through the 18-gauge tracheal cannula (n = 6–14 per group). BALF was then centrifuged at 1000 g for five minutes and the supernatant was extracted, snap frozen in liquid nitrogen, and stored at −80 °C. The supernatant was used for the determination of overall BALF protein concentration by Bio-Rad Protein Assay Dye Reagent (Bio-Rad). The supernatant was also utilized to evaluate the protein concentration of specific cytokines including IL-6, MIP-2, TNFα, and IL-1β by enzyme-linked immunosorbent assay (ELISA). Specific cytokine levels were quantitated by commercially available V-Plex MesoScale Discovery (MSD) ELISA kits (Meso Scale Diagnostics, Rockville, MD, USA). 

### 2.4. Immunohistochemistry for Inflammatory Cell Infiltration

Histologic analysis of inflammatory cell infiltrate was performed on lung tissue collected 24 h after infection (n = 6–7 per group). Lungs were inflated with melted 2% agarose solution and removed into 4% paraformaldehyde (PFA). The lung was immersion fixed in 4% PFA for 24 h at room temperature prior to dehydrating in 70% ethyl alcohol (EtOH). Dehydrated tissue was then embedded in paraffin and sectioned at 4 µm. Mounted sections were deparaffinized for immunohistochemistry with CD45 staining, a pan-leukocyte marker which stains all leukocytes brown under bright field microscopy. The Biocare Medical Decloaker was used to acquire the heat-induced epitope prior to slide staining with Leica’s Bond Rx instrument. Slides were then treated with primary CD45 antibodies (1:50 solution, BD Biosciences, Franklin Lakes, NJ, USA) and developed with Vectastain Elite ABC kit (Vector laboratories, Newark, CA, USA). Random imaging of ten high-powered fields (HPF) at 20x magnification was performed for each sample. The number of CD45-positive cells per HPF was quantified by a blinded researcher using an automated counting algorithm on NIS Elements—Advanced Research imaging software (Nikon Instruments, Melville, NY, USA). The 10 fields per sample were then averaged and compared between treatment groups.

### 2.5. Statistical Analyses

All statistical analyses were performed in GraphPad Prism 9.5.1 (La Jolla, CA, USA). Continuous variables were first tested for normality via Shapiro Wilk tests. The ROUT method was employed to identify and remove outliers. One-way ANOVA with multiple comparisons was then applied for normally distributed continuous variables with the following pairwise comparisons tested: PBS vs MRSA, PBS vs MRSA + CNP-miR146a, and MRSA vs MRSA + CNP-miR146a. The two-stage step-up method was used to control the false discovery rate [30]. The alpha value < 0.05 was considered statistically significant. 

## 3. Results

### 3.1. Intratracheal Treatment with CNP-miR146a

CNP-miR146a toxicity studies, including a 3-(4,5-dimethylthiazol-2-yl)-2,5-diphenyl tetrazolium bromide (MTT) toxicity assay, comparative pathology, and systemic distribution analyses, have been previously described [17]. Briefly, IT delivery of CNP-miR146a resulted only in upregulation of miR146a within the lungs while other tissues including kidney, liver, spleen, and heart-maintained baseline levels of miR146a for up to 168 h following IT delivery. Additionally, cerium concentration in plasma measured by ICP-MS was unchanged over 168 h after IT delivery of CNP-miR146a. 

### 3.2. MiR146a Expression Upregulated in CNP-miR146a Treated Mice

**Figure 1:**
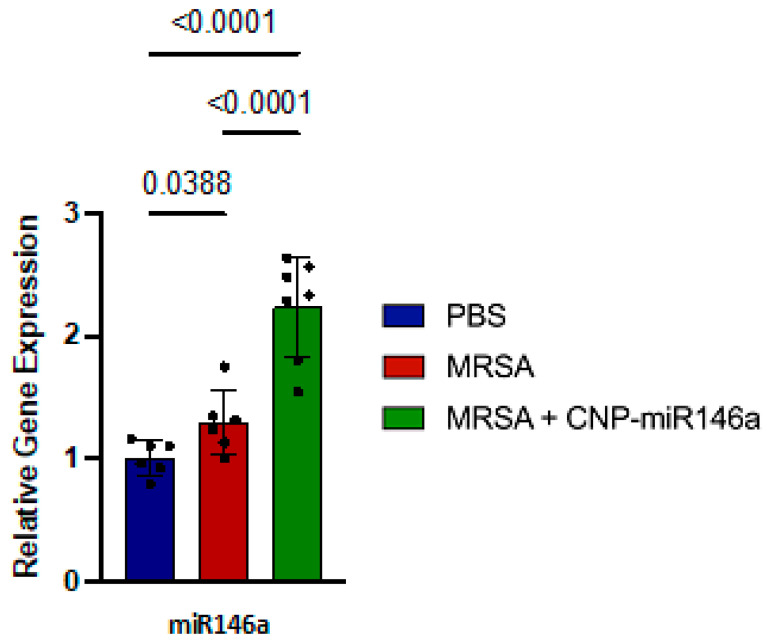
Relative gene expression of miR146a among study groups demonstrating significantly increased expression of miR146a in the CNP-miR146a treated group compared to both MRSA-infected lungs (*p* = 0.0001) and control (PBS) lungs (*p* < 0.0001).

Relative gene expression of miR146a between study groups was evaluated by collecting whole lung tissue 24 h after MRSA infection and processing it for RT-qPCR. MRSA-infected lungs, which were subsequently treated with CNP-miR146a, showed significantly increased expression of miR146a compared to both MRSA-infected and untreated (*p* < 0.0001) and control (PBS) lungs (*p* < 0.0001) (Figure 1). This is the expected result given miR146a is part of the conjugate therapeutic CNP-miR146a, and demonstrates effective delivery of the therapeutic to the target tissue. MiR146a was also increased in the MRSA-infected, untreated lungs compared to controls (*p* = 0.04) consistent with the natural pulmonary inflammatory response to an infectious insult; however, this increase was modest in comparison to the increase seen in the MRSA-infected, CNP-miR146a treated lungs and insufficient to prevent significant downstream inflammation. 

### 3.3. CNP-miR146a Decreases MRSA-Induced Proinflammatory Gene Expression

**Figure 2:**
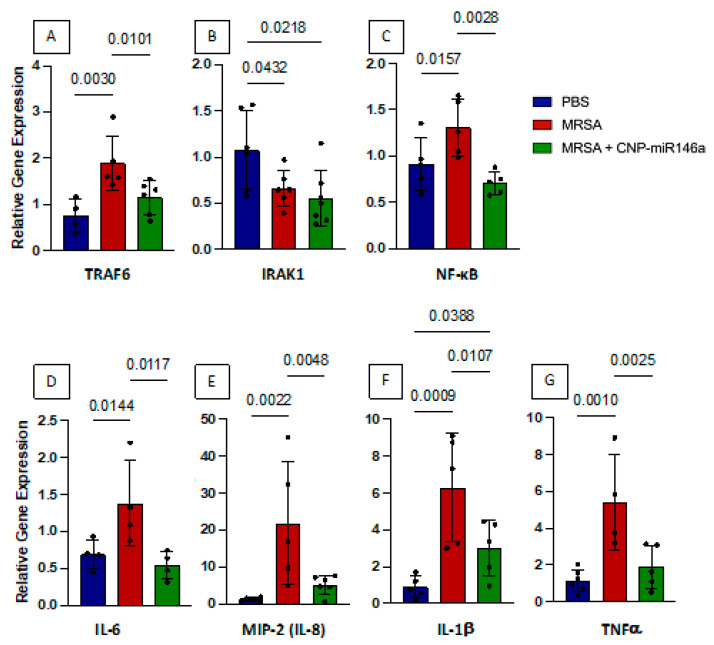
CNP-miR146a treatment four hours after infection significantly lowers proinflammatory gene expression. Relative gene expression compared to GAPDH of TRAF6 (**A**), IRAK1 (**B**), NF-κB (**C**), IL-6 (**D**), MIP-2 (**E**), IL-1β (**F**), and TNFα (**G**) 24 h after injury. MRSA infection resulted in significantly higher TRAF6, NF-κB, IL-6, MIP-2, IL-1β, and TNFα gene expression 24 h after injury. CNP-miR146a significantly lowered TRAF6, NF-κB, IL-6, MIP-2, IL-1β, and TNFα compared to untreated, MRSA-infected lungs. Control mice in blue, MRSA-infected and untreated mice in red, and MRSA-infected and CNP-miR146a treated mice in green.

Whole lung tissue was collected 24 h after infection and processed for RT-qPCR to evaluate for relative gene expression of proinflammatory genes IRAK1, TRAF6, NF-κB, IL-6, MIP-2 (murine IL-8 analog), IL-1β, and TNFα as these cytokines outline the pathway along which miR146a functions [22,23]. As depicted in Figure 2, MRSA infection significantly increased TRAF6 (*p* = 0.003), NF-κB (*p* = 0.02), IL-6 (*p* = 0.01), MIP-2 (*p* = 0.002), IL-1β (*p* = 0.0009), and TNFα (*p* = 0.001) relative gene expression 24 h after injury. Treatment with CNP-miR146a four hours after infection resulted in significantly decreased relative gene expression of TRAF6 (*p* = 0.01), NF-κB (*p* = 0.003), IL-6 (*p* = 0.01), MIP-2 (*p* = 0.005), IL-1β (*p* = 0.01), and TNFα (*p* = 0.003) compared to untreated, MRSA-infected lungs. No significant difference was seen between CNP-miR146a treated and control lungs with expression of TRAF6, NF-κB, IL-6, MIP-2, or TNFα, demonstrating that treatment with CNP-miR146a returned expression of these proinflammatory cytokines to control levels. IL-1β levels were decreased after CNP-miR146a treatment but were not restored to baseline levels. IRAK1 levels were higher in control lungs compared to both MRSA-infected and CNP-miR146a treated lungs. 

### 3.4. Improved Alveolar–Capillary Barrier Integrity with CNP-miR146a Treatment

**Figure 3:**
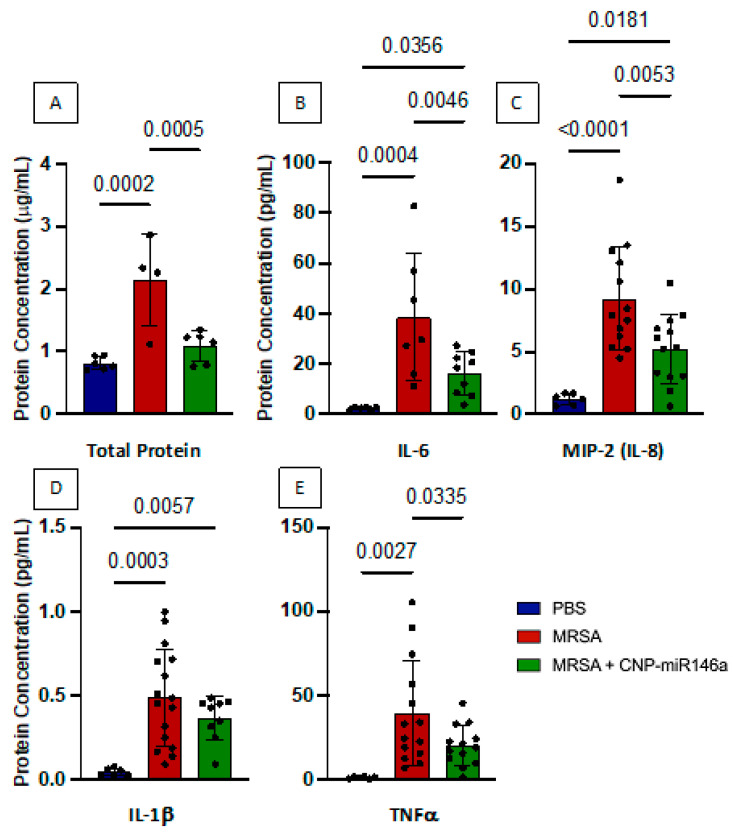
CNP-miR146a treatment of MRSA-induced ALI significantly lowers bronchoalveolar lavage fluid (BALF) proinflammatory protein concentration. BALF total protein concentration (**A**), and proinflammatory cytokines IL-6 (**B**), MIP-2 (**C**), IL-1β (**D**), and TNFα (**E**) were measured by ELISA 24 h after injury. MRSA infection resulted in significantly higher total protein, IL-6, MIP-2, IL-1β, and TNFα BALF protein concentration. CNP-miR146a significantly lowered total protein, IL-6, MIP-2, and TNFα compared to untreated, MRSA-infected lungs. Control mice in blue, MRSA-infected and untreated mice in red, and MRSA-infected and CNP-miR146a treated mice in green.

Total protein concentration in BALF was measured by BCA assay as a marker of alveolar–capillary barrier breakdown and protein leak. BALF total protein level was low in control mice indicating an intact alveolar–capillary barrier which prevents protein leak into the alveolar space. MRSA lung infection resulted in alveolar–capillary barrier breakdown as evidenced by significantly increased total protein concentration in BALF (*p* = 0.0002). IT treatment with CNP-miR146a four hours following MRSA-induced ALI resulted in significantly decreased BALF total protein (*p* = 0.0005) representing improved alveolar–capillary barrier integrity (Figure 3). Additionally, there was no difference in BALF total protein concentration between control mice and MRSA-infected, CNP-miR146a treated mice. 

Levels of proinflammatory cytokines IL-6, MIP-2 (murine IL-8 analog), IL-1β, and TNFα in BALF were measured by ELISA as physiologic markers of inflammation. Similar to total protein levels, control mice had very low BALF levels of all evaluated proinflammatory cytokines, consistent with uninjured and uninflamed lungs. BALF concentration of proinflammatory cytokines downstream of the NF-κB inflammatory pathway were significantly increased in MRSA-infected and untreated lungs including IL-6 (*p* = 0.0004), MIP-2 (*p* < 0.0001), IL-1β (*p* = 0.0003), and TNFα (*p* = 0.0003). CNP-miR146a treatment following MRSA lung infection resulted in significantly decreased IL-6 (*p* = 0.005), MIP-2 (*p* = 0.005), and TNFα (*p* = 0.03) in BALF. While BALF proinflammatory cytokine protein concentration was generally not returned to control levels as was seen with proinflammatory gene expression, there was significant reduction in BALF protein concentration in CNP-miR146a treated mice. 

### 3.5. CNP-miR146a Lowers Inflammatory Cell Infiltrate into the Lung

**Figure 4:**
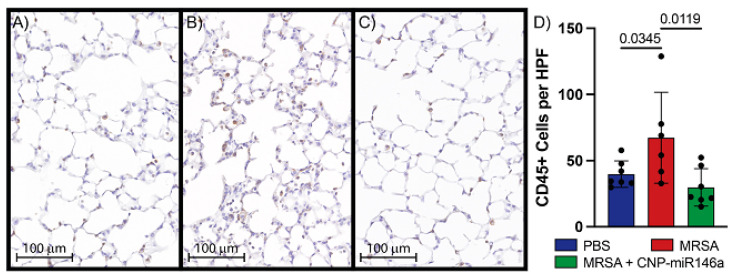
Treatment with CNP-miR146a significantly lowers histologic quantification of leukocyte infiltrate in the MRSA-infected lung. (**A**–**C**) 20× magnification slides of lung tissue stained for CD45 collected 24 h post-infection. Representative images of leukocyte infiltrate of (**B**) Control, (**C**) MRSA, (**D**) MRSA + CNP-miR146a. (**D**) Quantitative analysis of CD45+ cells per high-powered field (HPF). Control lungs in blue, MRSA-infected and untreated lungs in red, MRSA-infected and CNP-miR146a treated lungs in green.

Immunohistochemical analysis of leukocyte infiltrates into pulmonary tissue was performed on samples gathered 24 h after MRSA lung infection. Representative images at 20× magnification of slides stained for CD45+ cells and quantitative analysis of CD45+ cells per HPF are shown in Figure 4. There were significantly more CD45+ cells per HPF present in MRSA-infected and untreated lungs compared to controls (*p* = 0.03). Treatment with CNP-miR146a four hours following MRSA infection of the lungs significantly lowered leukocyte infiltrate compared to untreated, MRSA-infected lungs (*p* = 0.01, Figure 4D). There was no difference in CD45+ cell counts between control lungs and MRSA-infected, CNP-miR146a treated lungs suggesting CNP-miR146a returned inflammatory cell infiltrate to control levels. Qualitatively, control lungs appeared to have less alveolar collapse and debris than MRSA-infected lungs (Figure 4A,B), and although not quantified, the alveolar structure appeared subjectively preserved in the MRSA-infected lungs which had undergone treatment with CNP-miR146a (Figure 4C). 

## 4. Discussion

In the present study, we have shown that a single dose of CNP-miR146a four hours following pulmonary infection with MRSA significantly decreases proinflammatory gene signaling, bronchoalveolar proinflammatory protein leak, and inflammatory cell infiltrate compared to MRSA-infected and untreated lungs. Previously shown to decrease inflammation in both chemical (bleomycin) and inflammatory (LPS) lung injury models [17,18,19], this study highlights that IT delivery of CNP-miR146a also provides protection against inflammation in a clinically relevant infectious (MRSA) model of acute lung injury. 

We confirmed that instillation of IT MRSA induces a strong inflammatory response reflective of ALI and ARDS indicated by significantly increased inflammatory cell infiltrate and gene expression of proinflammatory cytokines IL-6, MIP-2, IL-1β, and TNFα as well as increased protein levels in BALF of these same proinflammatory cytokines, which is the functional physiologic output of inflammation. MRSA lung infection was also shown to alter alveolar–capillary barrier integrity as evidenced by elevated total protein concentration in BALF. IT treatment of MRSA-infected mice with CNP-miR146a mitigated the inflammatory response by decreasing proinflammatory gene expression leading to decreased concentrations of proinflammatory cytokines. While proinflammatory gene expression returned to control levels in CNP-miR146a treated lungs, BALF proinflammatory cytokine levels were not restored to baseline levels perhaps because gene expression precedes translation of protein, and the peak effect of treatment was not fully captured at this timepoint. CNP-miR146a also provided protection against structural degradation of the alveolar–capillary barrier with decreased protein movement into the BALF. 

Both components of the conjugated therapeutic, CNP and miR146a, have independent anti-inflammatory effects. Nanoceria have been shown to inhibit NF-κB leading to decreased levels of chemokines and cytokines significantly involved in leukocyte recruitment, such as IL-6 and IL-8 [12]. Additionally, miR146a directly inhibits TRAF6 and IRAK1, activators of NF-κB, which in turn indirectly downregulates the NF-κB inflammatory pathway similarly leading to decreased expression of proinflammatory cytokines [22,23]. While expected increases in TRAF6, NF-κB, and downstream proinflammatory cytokines were seen after MRSA infection compared to control, it remains unclear why a similar pattern was not demonstrated with IRAK1 relative gene expression. On histologic analysis, we see a robust infiltration of leukocytes in MRSA-infected lungs 24 h after inoculation, and a reduction in leukocyte infiltrate after treatment with CNP-miR146a (Figure 4). Neutrophil migration into the lungs has been clearly associated with ALI/ARDS severity [31,32], and thus a therapeutic that attenuates this process holds promise to reduce the clinical severity of ARDS. 

A complex network of cytokines play important roles in initiation, amplification, and maintenance of ALI and ARDS, with two of the most important early response cytokines being IL-1β and TNFα [33]. Additionally, IL-6 is an early acute phase reactant critical to the regulation of the acute-phase response to injury and infection [34]. Bronchoalveolar lavage (BAL) concentrations of IL-6, IL-8, IL-1β, and TNFα have been shown to be higher in non-survivors of ARDS, and increased IL-6 levels are correlated with oxygen index and more required ventilator days [31]. All of these cytokines are involved in the complex interplay of the proinflammatory feedback loop seen in ARDS. We demonstrated that treatment with CNP-miR146a following MRSA infection significantly reduced expression and production of IL-6, IL-8, IL-1β, and TNFα, which may function to prevent amplification of the positive feedback inflammatory cascade that is hallmark of ARDS. Early reduction in inflammatory gene expression may also serve to decrease pathologic remodeling.

Given the success of CNP-miR146a as a novel therapeutic for ALI and ARDS in now three different murine models representing a variety of ARDS etiologies, we are optimistic regarding the clinical utility of CNP-miR146a and its translation into the clinical setting. CNP-miR146a has a multitude of potential clinical applications including administration to intensive care unit patients with pneumonia, sepsis, COVID-19, or other risk factors for development of ARDS, administration prior to major abdominal surgery in patients with high risk of remaining intubated and mechanically ventilated post-operatively, and administration following major trauma in patients with significant pulmonary or thoracic injury, or those undergoing massive blood product transfusion. While IT instillation is the most feasible delivery route in murine models, we anticipate developing an inhaled route of therapeutic administration via inhaler or nebulizer for human use which could be given either prophylactically or after injury or infectious insult, both in a field or hospital setting. The promise of CNP-miR146a as a potential therapeutic is particularly exciting considering the limited current therapeutic options for ARDS. 

The results of our study must be interpreted in light of important limitations. While both male and female mice were utilized in this study, we did not perform analyses of any potential sex dimorphisms. MRSA pneumonia is not a natural pathogen in mice and is generally rapidly cleared by the host in murine models [35]. For this reason, an early timepoint was chosen for treatment and subsequent sample processing to capture the inflammatory response induced by this infectious model. Additionally, the rapid clearance of the pathogen limits evaluation of this model at later timepoints as the inflammatory response to MRSA inoculation is attenuated at later timepoints. Further study is warranted to quantify bacterial load and evaluate pulmonary function to determine the functional effects of targeting inflammation in the setting of infection, as decreased inflammation could be deleterious to bacterial clearance. 

In conclusion, we employed an infectious murine model of lung injury to test the efficacy of the novel anti-inflammatory therapeutic CNP-miR146a and utilized a variety of techniques to quantify its effect on inflammation. We found that treatment of MRSA-induced lung injury with CNP-miR146a reduced proinflammatory cytokine gene expression which correlated with reduced bronchoalveolar proinflammatory protein concentration and inflammatory cell infiltrate. This study shows the continued promise of CNP-miR146a as a potential therapeutic for the treatment of pediatric and adult ALI and ARDS. 

## Data Availability

The data presented in this study are available on request from the corresponding author.

## References

[1] Matthay M.A., Zemans R.L., Zimmerman G.A., Arabi Y.M., Beitler J.R., Mercat A., Calfee C.S. (2019). Acute respiratory distress syndrome. Nat. Rev. Dis. Primers.

[2] Cheung A.M., Tansey C.M., Tomlinson G., Diaz-Granados N., Matté A., Barr A., Mehta S., Mazer C.D., Guest C.B., Stewart T.E. (2006). Two-year outcomes, health care use, and costs of survivors of acute respiratory distress syndrome. Am. J. Respir. Crit. Care Med..

[3] Khemani R.G., Smith L.S., Zimmerman J.J., Erickson S. (2015). Pediatric acute respiratory distress syndrome: Definition, incidence, and epidemiology: Proceedings from the Pediatric Acute Lung Injury Consensus Conference. Pediatr. Crit. Care Med..

[4] Herridge M.S., Tansey C.M., Matté A., Tomlinson G., Diaz-Granados N., Cooper A., Cheung A.M. (2011). Functional disability 5 years after acute respiratory distress syndrome. N. Engl. J. Med..

[5] Kamdar B.B., Suri R., Suchyta M.R., Digrande K.F., Sherwood K.D., Colantuoni E., Dinglas V.D., Needham D.M., Hopkins R.O. (2020). Return to work after critical illness: A systematic review and meta-analysis. Thorax.

[6] Boucher P.E., Taplin J., Clement F. (2022). The Cost of ARDS: A Systematic Review. Chest.

[7] Acute Respiratory Distress Syndrome Network (2000). Ventilation with lower tidal volumes as compared with traditional tidal volumes for acute lung injury and the acute respiratory distress syndrome. N. Engl. J. Med..

[8] Parsons P.E., Eisner M.D., Thompson B.T., Matthay M.A., Ancukiewicz M., Bernard G.R. (2005). Lower tidal volume ventilation and plasma cytokine markers of inflammation in patients with acute lung injury. Crit. Care Med..

[9] Fielding-Singh V., Matthay M.A., Calfee C.S. (2018). Beyond Low Tidal Volume Ventilation: Treatment Adjuncts for Severe Respiratory Failure in Acute Respiratory Distress Syndrome. Crit. Care Med..

[10] Bellani G., Laffey J.G., Pham T., Fan E., Brochard L., Esteban A., Gattinoni L., Van Haren F., Larsson A., McAuley D.F. (2016). Epidemiology, Patterns of Care, and Mortality for Patients With Acute Respiratory Distress Syndrome in Intensive Care Units in 50 Countries. JAMA.

[11] Schwartz M.D., Moore E.E., Moore F.A., Shenkar R., Moine P., Haenel J.B., Abraham E. (1996). Nuclear factor-kappa B is activated in alveolar macrophages from patients with acute respiratory distress syndrome. Crit. Care Med..

[12] Selvaraj V., Nepal N., Rogers S., Manne N.D., Arvapalli R., Rice K.M., Blough E.R. (2015). Inhibition of MAP kinase/NF-kB mediated signaling and attenuation of lipopolysaccharide induced severe sepsis by cerium oxide nanoparticles. Biomaterials.

[13] D'Alessio F.R. (2018). Mouse Models of Acute Lung Injury and ARDS. Methods Mol. Biol..

[14] Kulkarni H.S., Lee J.S., Bastarache J.A., Kuebler W.M., Downey G.P., Albaiceta G.M., Altemeier W.A., Artigas A., Bates J.H.T., Calfee C.S. (2022). Update on the Features and Measurements of Experimental Acute Lung Injury in Animals: An Official American Thoracic Society Workshop Report. Am. J. Respir. Cell Mol. Biol..

[15] Vishnupriya M., Naveenkumar M., Manjima K., Sooryasree N.V., Saranya T., Ramya S., Winster S.H., Paulpandi M., Balachandar V., Arul N. (2021). Post-COVID pulmonary fibrosis: Therapeutic efficacy using with mesenchymal stem cells-How the lung heals. Eur. Rev. Med. Pharmacol. Sci..

[16] Jarczak D., Nierhaus A. (2022). Cytokine Storm-Definition, Causes, and Implications. Int. J. Mol. Sci..

[17] Niemiec S.M., Hilton S.A., Wallbank A., Azeltine M., Louiselle A.E., Elajaili H., Allawzi A., Xu J., Mattson C., Dewberry L.C. (2021). Cerium oxide nanoparticle delivery of microRNA-146a for local treatment of acute lung injury. Nanomedicine.

[18] Niemiec S.M., Hilton S.A., Wallbank A., Louiselle A.E., Elajaili H., Hu J., Liechty K.W. (2022). Lung function improves after delayed treatment with CNP-miR146a following acute lung injury. Nanomedicine.

[19] Wallbank A.M., Vaughn A.E., Niemiec S., Bilodeaux J., Lehmann T., Knudsen L., Smith B.J. (2023). CNP-miR146a improves outcomes in a two-hit acute- and ventilator-induced lung injury model. Nanomedicine.

[20] Heckert E.G., Karakoti A.S., Seal S., Self W.T. (2008). The role of cerium redox state in the SOD mimetic activity of nanoceria. Biomaterials.

[21] Kolanthai E., Fu Y., Kumar U., Babu B., Venkatesan A.K., Liechty K.W., Seal S. (2022). Nanoparticle mediated RNA delivery for wound healing. Wiley Interdiscip. Rev. Nanomed. Nanobiotechnology.

[22] Li L., Chen X.P., Li Y.J. (2010). MicroRNA-146a and human disease1. Scand. J. Immunol..

[23] Liu T., Zhang L., Joo D., Sun S.C. (2017). NF-κB signaling in inflammation. Sig. Transduct. Target. Ther..

[24] Rubinstein E., Kollef M.H., Nathwani D. (2008). Pneumonia caused by methicillin-resistant Staphylococcus aureus. Clin. Infect. Dis..

[25] Napolitano L.M., Brunsvold M.E., Reddy R.C., Hyzy R.C. (2009). Community-acquired methicillin-resistant Staphylococcus aureus pneumonia and ARDS: 1-year follow-up. Chest.

[26] Hayashida A., Bartlett A.H., Foster T.J., Park P.W. (2009). Staphylococcus aureus beta-toxin induces lung injury through syndecan-1. Am. J. Pathol..

[27] Zemans R.L., Matthay M.A. (2017). What drives neutrophils to the alveoli in ARDS?. Thorax.

[28] Neal C.J., Fox C.R., Sakthivel T.S., Kumar U., Fu Y., Drake C., Seal S. (2021). Metal-Mediated Nanoscale Cerium Oxide Inactivates Human Coronavirus and Rhinovirus by Surface Disruption. ACS Nano.

[29] Neal C.J., Sakthivel T.S., Fu Y., Seal S. (2021). Aging of nanoscale cerium oxide in a peroxide environment: Its influence on the redox, surface, and dispersion character. J. Phys. Chem..

[30] Benjamini Y., Krieger A.M., Yekutieli D. (2006). Adaptive linear step-up procedures that control the false discovery rate. Biometrika.

[31] Butt Y., Kurdowska A., Allen T.C. (2016). Acute Lung Injury: A Clinical and Molecular Review. Arch. Pathol. Lab. Med..

[32] Williams A.E., Chambers R.C. (2014). The mercurial nature of neutrophils: Still an enigma in ARDS?. Am. J. Physiol. Lung. Cell. Mol. Physiol..

[33] Crimi E., Slutsky A.S. (2004). Inflammation and the acute respiratory distress syndrome. Best Pract. Res. Clin. Anaesthesiol..

[34] Heinrich P.C., Behrmann I., Haan S., Hermanns H.M., Müller-Newen G., Schaper F. (2003). Principles of interleukin (IL)-6-type cytokine signalling and its regulation. Biochem. J..

[35] Sul C., Lewis C., Dee N., Burns N., Oshima K., Schmidt E., Nozik E. (2023). Release of extracellular superoxide dismutase into alveolar fluid protects against acute lung injury and inflammation in Staphylococcus aureus pneumonia. Am. J. Physiol. Lung. Cell Mol. Physiol..

